# Anaesthesia after neoadjuvant chemotherapy, immunotherapy or radiotherapy

**DOI:** 10.1016/j.bjae.2021.08.002

**Published:** 2021-10-16

**Authors:** M.D. Groenewold, C.G. Olthof, D.J. Bosch

**Affiliations:** 1 University Medical Centre Groningen, Groningen, the Netherlands; 2 Isala Zwolle, Zwolle, the Netherlands

**Keywords:** adverse effects, anaesthesia, cancer, neoadjuvant therapy, pharmacology, toxicity

**Learning objectives**

By reading this article, you should be able to:

• Discuss the advantages and disadvantages of prehabilitation and the importance of time to surgery.
• Describe the toxic adverse effects of neoadjuvant chemotherapy, radiotherapy and immunotherapy.
• Name the most commonly used chemotherapy agents and their indications.

**Key points**

• Prehabilitation improves physical performance and reduces complications.
• Extending time to surgery has negative consequences for survival from cancer and no benefits in reducing complications.
• Neoadjuvant chemotherapy is associated with toxic adverse effects on different organ systems.
• Neoadjuvant radiotherapy affects the targeted organ, but also has systemic effects.
• Neoadjuvant radiotherapy is associated with difficulties in airway management.

A significant number of patients with a solid tumour have to undergo neoadjuvant therapy comprising either chemotherapy, radiotherapy and immunotherapy (alone or in combination) before surgical resection of the tumour. Neoadjuvant therapy offers the ability to treat both the primary tumour and possible distant metastasis. These treatment modalities not only target rapidly dividing cancerous cells, but also non-malignant cells and thereby induce toxicity. Toxicity leads to serious adverse effects that can worsen a patient's health and is therefore relevant to the anaesthetist. Moreover, patients with liquid tumours, such as leukaemia or lymphomas, may present for surgery, for placement of vascular access or to treat complications of the treatment.

Toxic effects of neoadjuvant therapy can occur in different organ systems and may affect cardiac, pulmonary, renal, hepatic, bone marrow and immunological functions. Together with the patient's comorbidities and reduced nutritional status, a careful preoperative assessment is essential. Increasingly, prehabilitation is useful to improve a patient's functional capacity after neoadjuvant therapy, expedite recovery from the adverse effects of neoadjuvant therapy and recovery after surgery and reduce postoperative complications. Similarly, the time interval between neoadjuvant therapy and surgery plays an important role in physiological recovery, but is also an important determinant for survival from cancer.

This article aims to discuss the physiological effects of neoadjuvant chemotherapy, immunotherapy and radiotherapy, and also the preoperative considerations in terms of improving patient's condition in anticipation of surgery.

## Effect of chemotherapy on organ systems

### Immune response

Chemotherapeutic agents may have different effects on the immune system ranging from stimulatory to immunosuppressive effects. They affect both the innate and adaptive immune system. The innate immune system might be affected through myelosuppression leading to chemotherapy-induced neutropenia (CIN). If neutropenia is severe, the risks of systemic infections and sepsis increase. The incidence of neutropenia with fever (temperature >38.5°C) is around 50% for solid tumours and over 80% for haematological malignancies. The duration of neutropenia varies from a few days to weeks and depends on several factors, including the severity of neutropenia. In the management of CIN, multiple (irradiated) transfusions, myeloid growth factors (granulocyte colony-stimulating factor) to boost neutrophil production and antibiotics may be necessary.^1^ Pancytopenia, characterised by a reduction in the number of erythrocytes, leucocytes and platelets, may have serious consequences for perioperative care. During the perioperative period, oxygen-carrying capacity is decreased, whilst the risk of haemorrhage and opportunistic infection is increased. Preoperatively, haemoglobin concentration, thrombocyte counts and peripheral blood differentiation should be performed to exclude pancytopenia. In patients with (severe) CIN or pancytopenia, postponement of surgery should be considered until blood values have been recovered sufficiently.

Effects on the adaptive immune system may cause lymphopenia shortly after chemotherapy. Evidence about the degree of immunosuppression is contradictory and depends on chemotherapeutic agent and type of cancer. For example, alkylating agents and protein kinase inhibitors are related to an impaired proliferative or effector function of peripheral T-cells, whereas platinum-based chemotherapeutic drugs strongly enhance T-cell activation by dendritic cells (DCs).^2^

Cancer and neoadjuvant therapy both induce an inflammatory and procoagulant, antifibrinolytic and pro-aggregative response leading to venous thrombosis.^3^ The inflammatory response is responsible for a decreased concentration of anticoagulant proteins, including antithrombin and protein C. The procoagulant response is activated through increased tissue factor expression. The increased risk of thrombosis is sustained for up to 6 months with an increased perioperative risk of venous thromboembolic complications.^3^ Anticoagulation for venous thrombosis may interfere with or require careful timing for use of neuraxial techniques.

### Cardiovascular

Cardiotoxicity from chemotherapy occurs frequently and may evolve over a number of years. Although the pathophysiological mechanism is not fully understood, chemotherapeutic agents damage the myocytes in the heart, and as the heart has limited capacity to repair damage, these effects are prolonged, often presenting as left ventricular (LV) dysfunction, arrhythmias and heart failure (HF) (Table 1).

**Table 1:** Chemotherapeutic agents and associated toxic effects on different organ systems

Chemotherapeutic agent	Cardiovascular	Pulmonary	Renal	Hepatic	Other
**Alkylating agents** Cyclophosphamide Ifosfamide Melphalan Treosulfan Mitomycin C Busulfan	Myocardial ischaemia Heart failure Atrial fibrillation	ARDS Bronchospasm Interstitial pneumonitis Diffuse alveolar haemorrhage	AKI Proximal tubular injury Fanconi syndrome Nephrogenic DI	Sinusoidal injury Sinusoidal obstruction syndrome Centrilobular hepatocyte necrosis	Alopecia Cystitis Infections Peripheral neuropathy Bone marrow suppression Increased risk of thrombosis

**Anthracyclines** Doxorubicin Idarubicin	ECG changes Supraventricular arrhythmias Transient LV dysfunction Torsades de pointes Continuous decline in LVEF Chronic dilated cardiomyopathy Atrial fibrillation				Gastrointestinal effects Bone marrow suppression Increased risk of thrombosis

**Antimetabolites/pyrimidine antagonists** Gemcitabine Capecitabine Fluorouracil Tioguanine Fluoropyrimidines	Myocardial ischaemia Drug-induced thrombotic microangiopathy	Diffuse alveolar damage and haemorrhage Interstitial pneumonitis Capillary leak syndrome Pleural effusion	Oliguria SIADH	Steatosis	Bone marrow suppression Increased risk of thrombosis

**Arsenic trioxide**	QT prolongation Torsades de pointes Pericardial effusion			Severe hepatotoxicity in promyelocyte-treated patients	Nausea and vomiting Neuropathy Electrolyte disturbances Bone marrow suppression Increased risk of thrombosis

**Cytotoxic antibiotics** Bleomycin Mitomycin	Cardiomyopathy	Lung fibrosis			Mucositis Hyperkeratosis Gastrointestinal effects Bone marrow suppression Increased risk of thrombosis

**Platinum compounds** Carboplatin Cisplatin Oxaliplatin	Heart failure Atrial fibrillation Torsades de pointes	Interstitial pneumonitis	Acute kidney injury	Sinusoidal injury Sinusoidal obstruction syndrome Centrilobular hepatocyte necrosis	Peripheral neuropathy Infections Skin reactions Bone marrow suppression Increased risk of thrombosis

**Taxanes** Docetaxel Paclitaxel	Ventricular arrhythmias bradycardia Atrioventricular blocks	Interstitial pneumonitis Capillary leak syndrome			Peripheral neuropathy Infections Skin reactions

**Topoisomerase antagonists** Irinotecan Topotecan				Increased transaminases Steatosis Severe hepatocellular injury	Diarrhoea Nausea and vomiting Cholinergic syndrome

**Vinca alkaloids** Vincristine				Transient increase in transaminases	Peripheral neuropathy Bone marrow suppression Increased risk of thrombosis

AKI, acute kidney injury; ARDS, acute respiratory distress syndrome; DI, diabetes insipidus; LVEF, left ventricular ejection fraction; SIADH, syndrome of inappropriate antidiuretic hormone secretion.

Anthracyclines are associated with irreversible cardiac damage. The incidence of anthracycline-induced cardiotoxicity is 9–18% of patients regardless of restriction of the maximum cumulative dose. Early effects include ECG changes (non-specific ST and T-wave changes, decreased QRS voltage, prolongation of the QT interval, supraventricular arrhythmias and transient LV dysfunction).^4^ A chronic anthracycline-induced cardiomyopathy, caused by a continuous decline in left ventricular ejection fraction, may lead to chronic dilated cardiomyopathy.^4^

Myocardial ischaemia, infarction or ischaemia-induced arrhythmias are adverse effects of several classes of chemotherapy drugs (Table 1). Cancer therapy with 5-fluorouracil (5-FU) may lead to an incidence of almost 10% of myocardial ischaemia caused by coronary spasm and endothelial injury.^5^

Arrhythmias are frequently observed during or shortly after chemotherapy, with atrial fibrillation (AF) the most frequently observed supraventricular tachycardia (Table 1). Anthracyclines (2–10%), cisplatin (12–32%) and melphalan (7–12%) are all associated with AF.^6^ Other chemotherapeutic agents, especially arsenic trioxide, can cause QT prolongation that may lead to life-threatening arrhythmias (torsades de pointes).^7^

During preoperative screening, a focused history and examination, specifically exploring for signs and symptoms of cardiotoxicity, with consideration for a routine preoperative ECG (resting tachycardia, ST-segment and T-wave changes; conduction disturbances and QT prolongation; and arrhythmias) and laboratory tests (troponins and N-terminal pro-B-type natriuretic peptide) should be obtained (Table 2). If cardiotoxicity is suspected, patients should be seen by a cardiologist for further evaluation and medical optimisation.

**Table 2:** Preoperative and intraoperative management in patients treated with chemotherapy, immunotherapy or radiotherapy

Organ systems	Preoperative considerations	Intraoperative considerations
Cardiac	12-Lead ECG QT interval Echocardiography Laboratory tests: plasma troponin, NT-proBNP	5-Lead ECG Avoid drugs that prolong the QT interval Stress-dose glucocorticoids if indicated
Pulmonary	Baseline *S*po_2_ Chest X-ray Pulmonary function tests CT scan if indicated	Lung-protective ventilation Stress-dose glucocorticoids if indicated
Liver	Laboratory test: hepatic transaminases, INR	
Kidney	Laboratory tests: urea, creatinine, glomerular filtration rate	Balanced fluid management
Endocrinopathies (immunotherapy)	Laboratory tests: TSH, free T4, HbA_1C_, ACTH, cortisol, electrolyte balance Consultation with endocrinologist	Stress-dose glucocorticoids if indicated Balanced fluid management Electrolyte monitoring Possible interaction with antidepressants and antiemetic drugs
Head and neck (radiotherapy)	Airway management and planning	Difficult airway management
Others	Laboratory tests: basic tests, albumin Increased risk of thrombosis	Potential interaction with anticoagulant drugs

ACTH, adrenocorticotropic hormone; INR, international normalised ratio; NT-proBNP, N-terminal pro-B-type natriuretic peptide; TSH, thyroid-stimulating hormone.

### Pulmonary

Pulmonary toxicity can be induced by chemotherapy agents, of which bleomycin toxicity is the most commonly known. Life-threatening interstitial pulmonary fibrosis may develop in 5–16% of patients given bleomycin in concentrations >400 IU m^−2^.^8^ Lung injury typically develops within 6 months after the start of bleomycin treatment and is associated with life-long risks of pulmonary toxicity, especially when high inspired oxygen concentrations are used. Although it has recently been suggested that this incidence may be lower and reversible in most cases, it is recommended for safety that high inspired oxygen concentrations are avoided.^8^ In patients who are hypoxic, inspired oxygen should be titrated to achieve an *S*po_2_ between 88% and 92%.

In addition to bleomycin, alkylating agents may also lead to pulmonary toxicity. Mitomycin C can cause acute respiratory distress syndrome, bronchospasm and interstitial pneumonitis.^8^ Of the antimetabolites, gemcitabine is associated with diffuse alveolar damage and haemorrhage, interstitial pneumonitis, capillary leak syndrome with non-cardiogenic pulmonary oedema and pleural effusions (Table 1).^8^ Paclitaxel and docetaxel treatment may cause interstitial pneumonitis in hours to weeks after treatment in a minority (<5%) of patients.

Pulmonary toxicity can occur within weeks to months after the start of chemotherapy. Patients usually present with cough, followed by dyspnoea, hypoxaemia and low-grade fever. Physical examination often reveals bibasal crackles, and a preoperative chest X-ray will show unilateral or bilateral reticular markings, ground-glass opacities or consolidation (Table 2). High-resolution CT is sensitive, but not specific, and its prognostic value is unclear. Decreased diffusing capacity for CO_2_, total lung capacity and forced vital capacity are evident on pulmonary function testing. Chemotherapy-induced pulmonary toxicity is a diagnosis of exclusion and is treated with systemic glucocorticoids, stopping the causative agent and supportive measures.

### Kidney

Chemotherapeutic agents are often eliminated by the kidneys, and this can therefore lead to chemotherapy-induced acute kidney injury (AKI). Acute kidney injury can present with proximal tubular injury characterised by proteinuria, phosphate wasting, Fanconi syndrome (hypophosphataemia, hypokalaemia, glycosuria and proteinuria) and magnesium wasting (Table 1).

Cisplatin is one of the most widely known chemotherapeutic agents that can cause early development of AKI.^9^ One-third of patients will develop nephrotoxicity after cisplatin therapy with a consequent reduction in glomerular filtration rate (GFR), increased serum creatinine and reduced serum magnesium concentrations, which are mostly dose related and reversible.^10^ Proximal tubular injury, Fanconi syndrome and nephrogenic diabetes insipidus can be caused by the administration of ifosfamide (Table 1). In one study, renal failure developed in 80% of the patients who received ifosfamide 48 months after therapy, and two-thirds of patients developed Fanconi syndrome.^10^

Preoperative management and evaluation of the GFR and serum electrolytes are essential in patients exposed to nephrotoxic agents (Table 2). Patients with severe kidney dysfunction should be treated postoperatively with adjusted dosages of drugs excreted by the kidneys, for example some opioids, and a balanced approach to fluid management.

### Liver

Chemotherapeutic agents are mostly metabolised by the liver, and this is affected in patients with pre-existing liver disease. In most patients, hepatotoxicity is asymptomatic and is limited to increases in liver enzymes, but in severe cases, inflammatory hepatitis, cholestasis, steatosis and end-stage liver disease may occur. For instance, 5-FU may lead to steatosis, which is related to an increased risk of intraoperative blood loss and postoperative complications (Table 1). Multiple chemotherapeutics, such as oxaliplatin and busulfan, can cause hepatic sinusoidal injury, which can develop to sinusoidal obstruction syndrome.^11^

The National Cancer Institute and WHO have developed standardised criteria to grade the severity of chemotherapy-induced liver toxicity (Supplementary Table S1). Preoperatively, measurement of alkaline phosphatase, bilirubin, γ-glutamyl transpeptidase, alanine aminotransferase, aspartate aminotransferase and international normalised ratio can be used to evaluate liver function.

### Neurological effects

Neurotoxicity is a common adverse effect with many chemotherapeutic agents and is an important limiting factor in chemotherapy regimens. The development of neurotoxicity is dependent on cumulative dose and dose intensities, but is also more frequently seen in patients with diabetes mellitus, increased age, hereditary neuropathies or earlier treatment with neurotoxic chemotherapy. Chemotherapy may cause both peripheral and central neurotoxicity. Peripheral neurotoxicity affects predominantly sensory neurones and leads to peripheral neuropathy. Symptoms usually start during antineoplastic treatment and stabilise after treatment is completed. Central neurotoxicity may lead to a wide range of neurological disorders, such as encephalopathy, acute cerebellar syndrome, posterior reversible encephalopathy syndrome, aseptic meningitis, cognitive deficits, hemiparesis and progressive dementia.

Before surgery, a comprehensive neurological examination should be performed and documented in patients suspected of having neurotoxicity. In patients with peripheral neuropathy, attention should be paid to autonomic dysregulation, as this might lead to orthostatic hypotension. The use of regional anaesthesia is not contraindicated, but pre-existing neurological abnormalities should be documented.

## Effects of immunotherapy on organ systems

Cancer immunotherapies manipulate the host's immune system to recognise and reactivate the antitumour immune response. Different types of immunotherapies include interferon; immune checkpoint inhibitors (ICIs); and, more recently, chimeric antigen receptor T-cells. These genetically manipulated T-lymphocytes express T-cell receptors that can recognise tumour-specific antigens. Immune-related adverse reactions of immunotherapies relevant to perioperative care include endocrine, cardiac and pulmonary toxicities (Table 3).

**Table 3:** Immunotherapeutic agents and associated toxic effects on different organ systems

Immune checkpoint inhibitors and interferon	Cardiovascular	Pulmonary	Endocrine	Hepatic	Other
IpilimumabTremelimumabPembrolizumabNivolumabAtezolizumabAvelumabDurvalumab	Myocarditis Pericarditis Cardiac fibrosis Arrhythmias Heart failure Pericardial effusion	Pneumonitis	Hypophysitis Hypothyroidism Hyperthyroidism Primary adrenal insufficiency IDDM	Hepatic toxicity	Fever Fatigue Diarrhoea Nausea Anorexia Neuropsychiatric disorders Thrombocytopenia Leukopenia

IDDM, Insulin-dependent diabetes mellitus.

Immune checkpoint inhibitors may induce endocrinopathies, of which hypophysitis, an inflamed pituitary gland, is the most common.^12^ This condition can cause several clinical presentations, including hypothyroidism, adrenal insufficiency, hypogonadism and diabetes insipidus. Laboratory values of thyroid-stimulating hormone and adrenocorticotropic hormone, and electrolyte and acid/base balance should be examined during preoperative consultation (Table 2). Attention should also be paid to other endocrinopathies (hypothyroidism, hyperthyroidism, primary adrenal insufficiency and insulin-dependent diabetes mellitus) that can be caused by ICIs.^12^ If abnormalities are suspected, an endocrinologist should be consulted.

Toxic effects of immunotherapy on the cardiovascular system are rare, of which myocarditis is the most common (incidence of 1.14%). Clinical presentation ranges from non-specific (fatigue) to severe (dyspnoea and chest pain) symptoms. Patients with severe symptoms have increased serum brain natriuretic peptide and troponin concentrations and ECG abnormalities; these patients need consultation with a cardiologist.^13^ After diagnosis, ICIs are discontinued and patients need to be treated with high-dose glucocorticoids, which might need to be continued at a ‘stress’ dose during perioperative care. Other cardiac adverse effects associated with immunotherapy include pericarditis, cardiac fibrosis, arrhythmias and HF.^13^

Pulmonary toxicity induced by ICIs is mostly limited to pneumonitis. Although the incidence is low, the presentation is potentially life-threatening, but may range from cough, chest pain, wheezing, shortness of breath to respiratory failure. To distinguish from other pulmonary comorbidities, a CT scan should be made and a consultation with the pulmonologist is recommended. The management of patients with pneumonitis consists of antibiotics and glucocorticoids, which must be converted to a stress dose during perioperative care.

Because there is a wide variety of ICIs, the anaesthetist should pay attention to specific agents and toxicities. For a detailed overview of anaesthetic considerations in patients treated with immunotherapy, the reader is recommended to read recent narrative reviews on perioperative implications of novel anticancer therapies.^12^^,^^14^

## Effects of radiotherapy on organ systems

### Immune system

Irradiation affects the immune system, which can be observed both inside and outside the tumour. DNA damage-induced cell apoptosis releases alarmins, which activate the innate and adaptive immune system that results in a pro-inflammatory response. This response activates T-cells to induce a systemic antitumour inflammatory response, the abscopal effect. It is unclear whether this proinflammatory environment with activated cytokines, chemokines and other damage-associated molecular patterns (DAMPs) has consequences for the perioperative period. Conversely, irradiation also leads to immunosuppression by inactivating natural killer cells and DCs.^15^

The pro-inflammatory immune response of radiation also affects the vascular endothelium. In mild doses of 5–10 Gy per fraction, vascular endothelial damage is relatively mild, whereas higher doses above 10 Gy per fraction cause considerable damage with increased vascular permeability. This is caused by apoptosis of different cell layers in the vessel wall, which leads to increased risk of thrombosis because of platelet aggregation and fibrosis.

### Cardiovascular

Radiation toxicity to the heart results from a combination of endothelial damage, oxidative stress with inflammation and genetic (mitochondrial DNA) damage. Irradiation with a sufficiently high dose can damage any component of the heart, including the pericardium, myocardium, heart valves, coronary arteries, capillaries and conducting system (Table 4). Exudative pericarditis can develop at an early stage and may be accompanied by haemodynamic abnormalities, but is generally self-limiting. Abnormalities in the conducting system are another early complication and can occur within months, but are usually self-limiting after 12 months. Different rhythms can be seen, such as atrioventricular block, QTc prolongation, supraventricular arrhythmia and ventricular tachycardia (Table 2). Coronary artery disease is a late consequence of radiation therapy.

**Table 4:** Toxic effects of radiotherapy on different organ systems

Cardiovascular	Pulmonary	Head and neck	Other
Pericarditis Arrhythmias Pericardial effusion QT interval prolongation Coronary artery disease	Pulmonary oedema Pneumonitis Lung fibrosis	Mucositis Dry mouth Altered taste Dermatitis Osteonecrosis Trismus Dysphagia Changes in airway	Hypothyroidism

ACTH, adrenocorticotropic hormone; INR, international normalised ratio; NT-proBNP, N-terminal pro-B-type natriuretic peptide; TSH, thyroid-stimulating hormone.

### Pulmonary

Lung injury from radiation therapy can be divided into three phases: acute, subacute and late radiation toxicity. The acute phase occurs within hours or days after radiation therapy, caused by an inflammatory response and direct DNA damage. In response, DAMPs and reactive oxygen species are released, leading to further damage of (mitochondrial) DNA, pulmonary oedema, increased vascular permeability and eventually apoptosis of alveolar Type I pneumocytes.^16^ These inflammatory changes can lead to pneumonitis in the subacute phase (2–6 months after irradiation) depending on the mean lung dose, the proportion of the lung volume receiving >20 Gy of radiation and underlying comorbidities. In most patients, it is limited to an asymptomatic increased density on CT scan, but others develop a sterile pneumonitis, including a non-productive cough, dyspnoea and occasionally low-grade fever. In patients with severe symptoms, glucocorticoids might be needed. Surgery will often coincide with the sub-acute phase. Nevertheless, there is no recent literature describing an increased risk for perioperative pulmonary complications after radiotherapy. Late radiation toxicity occurs after 9–12 months and involves an irreversible remodelling of lung parenchyma with increasing stiffness and thickening from fibrosis.

### Head and neck

Radiotherapy for head and neck cancers is associated with debilitating adverse effects. In the acute phase, defined within weeks, patients may complain about mucositis, dry mouth, altered taste, dermatitis, osteonecrosis, trismus and dysphagia (Table 4). Moreover, if radiotherapy is targeting the neck, patients may develop hypothyroidism or difficulties with airway management (Table 2). Thyroid status should be evaluated before surgery in patients presenting with symptomatic signs of hypothyroidism after radiotherapy. Surgery in patients with moderate or severe hypothyroidism should be delayed until hypothyroidism has been corrected.

The anaesthetist needs to be aware of difficulties with airway management in patients treated with radiotherapy for head and neck cancers. Difficulties may arise in mask holding and ventilation because of osteonecrosis, mucositis, lack of dentition, decreased mobility of the neck and oedema from radiation. Laryngoscopy may be difficult because of fibrosis, trismus and restrictions in mouth opening, and decreased mobility of the tongue and neck. Glottic and epiglottic oedema may impede visualisation of normal anatomy. Careful preoperative airway assessment and planning, including ability to perform awake fibreoptic intubation, are essential.

## Preoperative management

As part of the preoperative assessment, the anaesthetist should note the patient's neoadjuvant therapy and possible toxic effects reported. Physical examination should include an accurate evaluation of cardiopulmonary status, combined with ECG, pulse, baseline *S*po_2_, arterial pressure and laboratory tests (Table 2). These may reveal clinical features of adverse effects that require further investigation. In some patients taking neoadjuvant agents associated with common systemic or organ-specific toxicities, further investigations, such as cardiopulmonary imaging or pulmonary function testing, may be needed to inform preoperative assessment and choice of anaesthetic technique. It may be necessary to delay surgery until critical organ dysfunction is recovered. Difficulties with airway management should be anticipated in patients treated with radiotherapy for head and neck cancers; careful airway assessment and planning are required. Specific considerations for preoperative optimisation are discussed next.

### Timing of surgery

It is generally advised that time to surgery (TTS) after neoadjuvant therapy should be several weeks to allow a reasonable recovery from cytotoxic adverse effects and a reduction in disease volume, thereby optimising conditions for surgical resection. A TTS of 4–6 weeks is often considered the optimal interval to achieve these goals, although in many cases, the optimal timing is unknown. Different trials have evaluated the consequences of a prolonged TTS, partly during the COVID-19 pandemic, but evidence suggests that delayed resection worsens survival.^17^^,^^18^ However, a longer TTS may allow greater recovery from the toxic effects of neoadjuvant therapy before surgery. In an RCT in patients with oesophageal cancer, no differences in short-term postoperative outcomes were found between standard (4–6 weeks) or prolonged (10–12 weeks) TTS.^19^ A standardised TTS of 4–6 weeks after completion of neoadjuvant therapy mandates timely access to effective prehabilitation.

### Prehabilitation

Prehabilitation aims to improve a patient's functional status before surgery and enhance postoperative recovery. Studies suggest that prehabilitation improves physical performance and quality of life, and decreases postoperative complications and hospital length of stay.^20^^,^^21^ Different forms of prehabilitation have been introduced over the past decade, but a multimodal, individualised approach focusing on nutritional, psychological and physical assessments and intervention seems to provide the best results.^21^

#### Nutrition

The Enhanced Recovery After Surgery guidelines identified poor nutritional state as a risk factor for postoperative complications, whilst preoperative optimisation of nutritional status could reduce these risks significantly. For various reasons, patients who have undergone neoadjuvant therapy are at risk of nutritional deficiencies. A growing tumour with increasing metabolic demands may result in a disturbed metabolism. In addition, patients with tumours involving the gastrointestinal tract may be unable to process and digest food. Furthermore, chemotherapeutic agents can have adverse effects, such as nausea, emesis or mucositis, leading to diarrhoea. Nutritional intake and weight loss should be evaluated at the preoperative assessment. Nutritional risk scores can be helpful (e.g. NRS-2002 or Riley's nutritional risk screening, which correlates with postoperative mortality or morbidity). When malnutrition is suspected, a dietician should be consulted and laboratory tests for albumin, kidney function and electrolytes; abnormalities should be corrected when needed.

#### Psychological support

Before surgery, patients may suffer from anxiety, depression and low self-efficacy, which is associated with worse physiological surgical outcomes and postoperative quality of life. Physiological interventions, such as education programmes, may reduce anxiety and increase a patient's knowledge and understanding. Procedural and sensory information is an essential part of the preoperative assessment and proven beneficial effects on postoperative pain and recovery.^22^ The cessation of smoking and excessive alcohol consumption must be addressed; intensive counselling combined with nicotine replacement therapy has proved to be the most effective way to achieve this.

#### Exercise training

Cancer and neoadjuvant therapy result in a decline in fitness from skeletal muscle wasting and cachexia. Different exercise programmes have been developed, such as inspiratory muscle training and aerobic or cardiopulmonary exercise training. Inspiratory muscle training consists of an inspiratory threshold device to increase the strength and endurance of inspiratory muscles. This could decrease postoperative pulmonary complications caused by alveolar collapse, which occurs frequently in patients with respiratory muscle weakness. Aerobic or cardiopulmonary exercise training consists of ergometric cycle training sessions several times a week to achieve an improvement in anaerobic thresholds. On theoretical basis, maximum results of prehabilitation might be achieved in the elderly or frail patients, but different results have been described. In an RCT of patients with frailty, preoperative aerobic training did not result in reductions in complications or length of stay after colorectal surgery.^23^ Conversely, an RCT that included high-risk patients for major abdominal surgery reported a 50% reduction in postoperative complications in the intervention group.^24^

Based on these data, it seems justified to integrate a multimodal prehabilitation programme into the interval between neoadjuvant therapy and surgery. However, further research is needed into which patients benefit and the most effective form of prehabilitation.

## Intraoperative management

In addition to standard intraoperative monitoring, patients may require invasive monitoring because of chemotherapy-induced toxicity or in cases of extensive surgery or blood loss, where haemodynamic fluctuations are anticipated (Table 2). Additional considerations include the presence of pancytopenia, which increases the risk of perioperative haemorrhage. Perioperative transfusion of blood products is associated with worse oncological outcomes, probably related to impaired immunological function. In addition to transfusion of blood products, alternatives should be considered, including desmopressin and antifibrinolytic agents. To prevent the complications of blood product transfusion, leucocyte-reduced and irradiated blood components should be considered in patients who are immunocompromised. Despite fears of further spread of malignant cells, intraoperative cell salvage in combination with leucodepletion filters is considered to be safe during oncological surgery. Furthermore, optimal temperature regulation is not only pivotal for controlling haemorrhage, but also for reducing infection.

In specific circumstances, there might be a preference for general or regional anaesthesia in the management of a patient with toxicity from neoadjuvant therapy. Neuraxial techniques can provide optimal postoperative pain relief, especially in patients with long-term opioid consumption, but might also decrease cardiopulmonary risk in patients with neoadjuvant therapy toxicity. Precautions, such as an increased risk of bleeding in patients with thrombocytopenia and peripheral neuropathy after chemotherapy, should be considered. There has been a debate for several years about the effects of anaesthetic technique on long-term survival, which is beyond the scope of this review. In general, it has been suggested that regional anaesthesia has beneficial effects compared with general anaesthesia alone, and that propofol improves oncological survival compared with volatile anaesthesia. Despite many studies and reviews, there is still no consensus on the optimal anaesthetic technique for long-term survival after oncological surgery.

As different chemotherapeutic regimens and thoracic radiotherapy are associated with pulmonary toxicity, lung-protective ventilation (inspiratory plateau pressures <30 cm H_2_O and tidal volumes normalised to predicted body weight) is recommended (Table 2). Arterial blood gas analysis during surgery is helpful. Patients treated with bleomycin have a life-long risk of pulmonary toxicity, especially when high inspired oxygen concentrations are used. Anaesthetists need to be aware of difficulties with airway management in patients treated with radiotherapy for head and neck cancers.

## Summary

Neoadjuvant therapy consisting of either chemotherapy, radiotherapy or immunotherapy is associated with toxic effects on different organ systems. These toxic effects may interfere with perioperative care, but may also cause a deterioration in the patient's general condition to such an extent that the risk of perioperative complications increases. During preoperative assessment, a structured history, physical examination and additional examinations should be performed to detect toxic adverse effects from neoadjuvant therapy, and optimisation before surgery is essential. Multimodal prehabilitation before surgery seems to lower these risks.

## Declaration of interests

The authors declare that they have no conflicts of interest.

## MCQs

The associated MCQs (to support CME/CPD activity) will be accessible at www.bjaed.org/cme/home by subscribers to *BJA Education*.

## Supplementary Material

Supplementary material can all be viewed from the article in *BJA Education* online.
