# Supplementary material for: Anaesthesia after neoadjuvant chemotherapy, immunotherapy or radiotherapy
Source: BJA Educ. 2021 Oct 16;22(1):12–9. doi: 10.1016/j.bjae.2021.08.002 (PMC8703124; doi:10.1016/j.bjae.2021.08.002)
Supplement: Multimedia component 1 [file mmc1.docx]

|  | Grade1 | Grade 2 | Grade 3 | Grade 4 | Grade 5 |
| --- | --- | --- | --- | --- | --- |
| Alkaline phosphate | > ULN -2,5x ULN | > 2,5-5 ULN | > 5-20 ULN | > 20 x ULN |  |
| Bilirubin | > ULN -1,5x ULN | > 1,5-3 ULN | > 3-10 ULN | > 20 x ULN |  |
| Y glutamyl transpeptidase (GGt) | > ULN -2,5x ULN | > 2,5-5 ULN | > 5-20 ULN | > 20 x ULN |  |
| Aspartate aminotransferase (AST) | > ULN -3x ULN | > 3-5 ULN | > 5-20 ULN | > 20 x ULN |  |
| Alanine aminotransferase (ALT) | > ULN -3x ULN | > 3-5 ULN | > 5-20 ULN | > 20 x ULN |  |
| Liver Failure |  |  | Asterixis, mild hepatic encephalopathy (HE) | Moderate-severe HE; life-threatening consequences | Death |
| Portal Hypertension |  | Decreased portal vein flow |  | Life-threatening consequences; urgent operative intervention needed | Death |

National Cancer Institute terminology for hepatotoxicity

Abbreviation: ULN, upper limit of normal

Tabel uit: Drug-induced liver injury due to cancer chemotherapeutic agents. Bahirwani R, Reddy KR, Semin Liver Dis 2014;34:162-171
